# Transglutaminase 3: The Involvement in Epithelial Differentiation and Cancer

**DOI:** 10.3390/cells9091996

**Published:** 2020-08-30

**Authors:** Elina S. Chermnykh, Elena V. Alpeeva, Ekaterina A. Vorotelyak

**Affiliations:** Koltzov Institute of Developmental Biology Russian Academy of Sciences, 119334 Moscow, Russia; alpeeva_l@mail.ru (E.V.A.); vorotelyak@yandex.ru (E.A.V.)

**Keywords:** transglutaminase, hair follicle, epidermis, cornification, carcinoma

## Abstract

Transglutaminases (TGMs) contribute to the formation of rigid, insoluble macromolecular complexes, which are essential for the epidermis and hair follicles to perform protective and barrier functions against the environment. During differentiation, epidermal keratinocytes undergo structural alterations being transformed into cornified cells, which constitute a highly tough outermost layer of the epidermis, the stratum corneum. Similar processes occur during the hardening of the hair follicle and the hair shaft, which is provided by the enzymatic cross-linking of the structural proteins and keratin intermediate filaments. TGM3, also known as epidermal TGM, is one of the pivotal enzymes responsible for the formation of protein polymers in the epidermis and the hair follicle. Numerous studies have shown that TGM3 is extensively involved in epidermal and hair follicle physiology and pathology. However, the roles of TGM3, its substrates, and its importance for the integument system are not fully understood. Here, we summarize the main advances that have recently been achieved in TGM3 analyses in skin and hair follicle biology and also in understanding the functional role of TGM3 in human tumor pathology as well as the reliability of its prognostic clinical usage as a cancer diagnosis biomarker. This review also focuses on human and murine hair follicle abnormalities connected with TGM3 mutations.

## 1. Introduction

**Figure 1:**
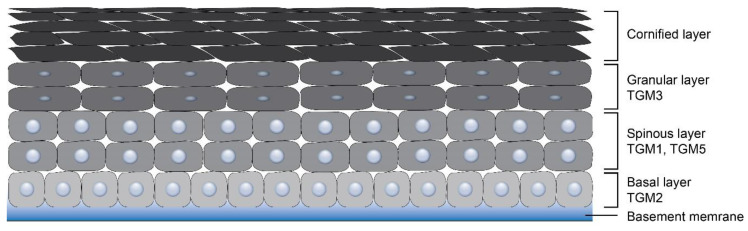
Distribution of transglutaminases (TGMs) in the epidermis.

Many physiologically important protein cross-linking reactions in mammals are orchestrated by transglutaminases (TGMs). These enzymes catalyze the formation of the protein network by introducing isopeptide bonds between lysine and glutamine residues of the target proteins [1,2]. TGMs are also engaged in the deamidation reaction of glutamine residues, the covalent conjugation of polyamines and the lipid esterification [3,4]. The primary purpose of TGM-mediated posttranslational protein modification is the formation of stabilized and insoluble protein polymer structures, such as a cornified cell envelope (CCE), a hair fiber, bones, or a fibrin clot [2]. Some TGMs may function as atypical GTPases and ATPases, protein disulfide isomerases, protein kinases, and also have non-enzymatic functions associated with cell signaling and cell–matrix interactions [5,6]. Depending on the distribution in organs and tissues, TGMs are classified into separate groups known as TGM types. In humans, nine types of TGMs, TGM1-7, Factor XIII and Band 4.2, have been documented and characterized. These enzymes fulfill a variety of physiological functions and are related to different pathological processes [2,6]. All mammalian TGMs have structural homology, although they can display the differences in their N- and C-terminal domains [7,8]. All TGMs except Band 4.2 were found to be expressed in the integument system at the mRNA level [9,10]; however, the contribution to the skin development of three of them (TGM4, TGM7, Factor XIII) remains to be investigated. TGM4 is thought to be exclusively involved in prostate gland morphogenesis and is present in the seminal plasma [11]. The function of TGM7, showing widespread distribution in different tissues with high expression pattern restricted to testes and lungs, remains unclear [12]. Factor XIII was unequivocally confirmed to play a role in the fibrin clot stabilization [13], but recent studies suggest it to be involved in corneal epithelium maintenance and corneal wound healing process [14]. As for the Band 4.2, it is related to the regulation of the shape and mechanical properties of erythrocytes [15]. The remaining five TGMs are determined to be of great importance for epidermal and hair follicle morphogenesis (Table 1) playing mainly a structural role and are distributed in all epidermal layers (Figure 1). Mutations in some of them result in dermatological pathologies. TGM1 makes a predominant contribution to CCE formation. The inborn error in TGM1 gene in mice results in an absence of CCE and impaired skin barrier function [16]. TGM2, in contrast, is thought to play a minor role in keratinocyte CCE assembly, since mice lacking TGM2 are viable, phenotypically normal, and do not show skin barrier defects [17]. According to some authors, TGM2 is constitutively expressed in all epidermal layers [18]; however, other authors were able to detect TGM2 only in basal keratinocytes under specific conditions, such as wound healing and repair (Figure 1) [19]. Although TGM2 does not seem to play the central role in the epidermis, it has been recently revealed as a mediator of the epidermal inflammatory response to UV irradiation [18]. TGM2 is also localized in dermal fibroblasts and contributes to extracellular matrix formation via binding fibronectin and collagens together [20]. TGM2 is found in numerous tissues and is implicated in multiple processes, including wound healing, proliferation, apoptosis, cell adhesion, and migration [21,22]. TGM5 plays an important role in keratinocyte differentiation and the cornification of the epidermis, since its epidermal expression is altered in diseases related to abnormal keratinization, such as psoriasis and ichthyosis [23]. TGM6 is associated with neuronal differentiation [24], but its high expression was also detected in the skin [25]. TGM6 was mapped in close proximity to TGM3 on chromosome 20 in humans and shares more than a 50% sequence similarity to it. The donor substrates of TGM6 and Ca^++^-binding sites are similar to those of TGM3 [25]. This signature allows us to suggest TGM6 involvement in epidermal differentiation. TGM1, TGM3, TGM5, and TGM6 are widely expressed in epidermal keratinocytes being mainly responsible for the cross-linking of proteins to form CCE in the skin epidermis and to strengthen hair fiber [26,27,28].

The main focus of this review is TGM3, also referred to as epidermal TGM. This enzyme was initially discovered in the hair fiber and hair follicle [29,30], and was subsequently found in the epidermis, mucosa, brain, stomach, spleen, small intestine, testes, and skeletal muscles [31,32,33]. In the skin and mucosa, TGM3 is expressed in the suprabasal layers of the stratified squamous epithelium [34]. TGM3 was shown to participate in different processes relating to hair follicle morphogenesis, and together with TGM1 contributes to human hair abnormalities. Although the expression of TGM3 was detected in many other organs, the biological function of TGM3 is well described only for stratified squamous epithelium and has not been sufficiently investigated in other epithelial tissues. The reports mainly document the involvement of TGM3 in different pathologies. Some authors suggest its role as a tumor suppressor and its involvement in apoptosis [33,34,35,36]. Either the downregulation or upregulation of TGM3 correlates with malignant transformation of epithelia. The potential involvement of TGM3 in diverse biological processes makes it a subject of special interest for scientists.

Below we address the physiological roles of TGM3 in the keratinization processes in the epidermis and hair follicle and consider the contribution of TGM3 to different pathologies not only related to hair.

## 2. Contribution of TGM3 and Other TGMs to Skin Morphogenesis

### 2.1. Epidermal Cornification

Epidermal cornification is a unique form of terminal differentiation and the programmed cell death of epidermal keratinocytes. Through their lifespan, keratynocytes undergo complex transformation, at the initiation stage of which basal keratinocytes exit the cell cycle and start migrating from the basement membrane through the spinous and granular layers outward to the surface of the skin. While migrating, they modify their transcriptional program to produce necessary structural proteins and enzymes specific to the current cell position within the skin [46,47]. Cornification is characterized by the accumulation of densely packed keratin intermediate filaments (KIFs) in the cytoplasm, the elimination of nuclei and other organelles, and the substitution of cell plasma membrane by insoluble protein–lipid matrix, named CCE. The assembling of this structure is gradual. It begins while maturing keratinocytes move through the spinous layer and is completed when the cells reach the outer cornified layer (stratum corneum) [46,47]. Thus, the stratum corneum consists of flattened, dead keratinocytes called corneocytes embedded in a lamellar lipid–protein matrix. CCE gives the cornified layer flexible mechanical resistance and provides a scaffold for the formation of intercellular corneodesmosome links and the extracellular lipid–protein matrix [48].

The activation and expression of TGM1, TGM3 and TGM5, which are present in the epidermis (Figure 1), have been proven to be implicated to varying degrees in CCE formation and various other proteins contribute to this process. First, TGM1 and TGM5 become activated in the spinous layer for the cross-linking of envoplakin and periplakin to the desmosomes under the cell membrane [28,49]. Moving farther into the granular layer, keratinocytes synthesize markers of later differentiation—involucrin, loricrin, filaggrin, and small proline-rich proteins (SPRs). Filaggrin is biosynthesized as profilaggrin. It organizes KIFs into tight, oriented bundles and is accumulated in the form of keratohyalin granules. At the later stage, profilaggrin undergoes dephosphorylation and is proteolytically converted to filaggrin. KIFs are cross-linked by isopeptide bonds to CCE, primarily through a single lysine residue located on the head domain of the type II keratin chains [50], while a very low amount of filaggrin is covalently linked to CCE [51]. Substrates, such as SPRs and loricrin, are cross-linked by TGM3 in the cytoplasm and then translocated to growing scaffold on cell periphery, where TGM1 additionally cross-links them to already existing protein scaffold on a plasma membrane to complete the process of CCE formation [52]. Involucrin molecules become cross-linked by TGM1 on a plasma membrane to involucrin itself and also to loricrin, desmoplakin, periplakin, and envoplakin. A protein scaffold accumulates under the plasma membrane and significantly strengthens it. Membrane-bound TGM1 cooperatively with TGM5 also catalyzes cross-linking between involucrin and ceramids, the main lipid element of the stratum corneum [53]. This reaction connects the inner protein component of CCE to the outer lipid one.

TGM3 is expressed as an inactive zymogen that must undergo proteolytic processing for fulfilling its enzymatic functions. For the promotion of cross-linking activity, the zymogen should be released into two fragments of 47 and 30 KDa during differentiation, which remain associated together in the active enzyme [54,55]. It has been suggested that cathepsin L released from the degraded lysosomes can cleave the TGM3 zymogen to produce an active enzyme [55]. The in vitro studies also revealed that dispase, proteinase K, trypsin, and thrombin [56] are able to switch TGM3 from an inactive to active form [57].

The binding of Ca^++^ ions is important for TGM3 to exert its cross-linking activity after it has been cleaved by the protease [8]. There is a gradient of Ca^++^ concentration in the epidermal layers of the skin. In the basal layer, Ca^++^ concentration is very low, which supports the undifferentiated state of keratinocytes. In the spinous layer, the concentration of Ca^++^ is growing, reaching the maximum in the granular layer and enabling the maximal enzymatic activity of TGM3 [58,59,60].

Overall, TGM3 has a distinct role in protein cross-linking and builds CCE cooperatively with TGM1 and TGM5, which all act in a highly coordinated manner.

### 2.2. Hair Keratinization

The mature hair follicle has a complex organization and undergoes a specialized cornification process in the hair cycle. It is accompanied by the specific expression of various keratins and associated proteins, which bind together through enzymatic protein modification producing the stabilized and hard hair shaft.

**Figure 2:**
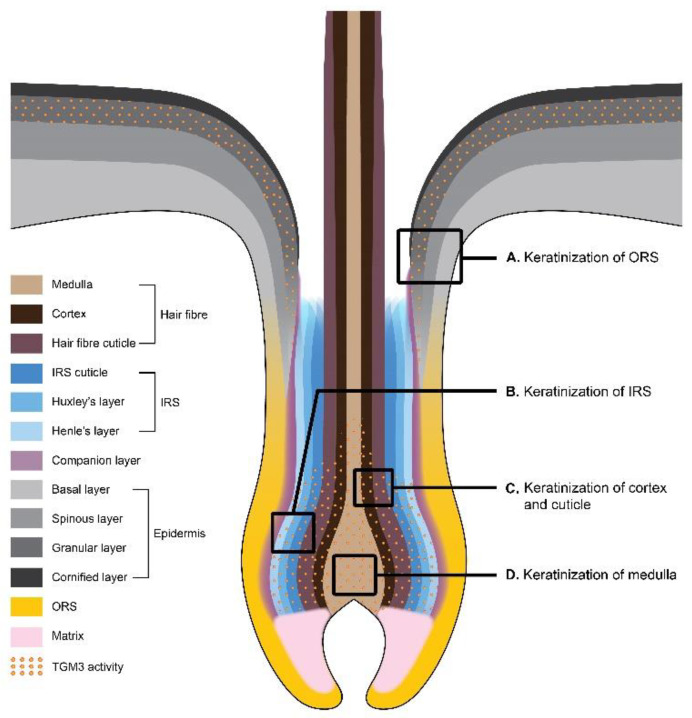
Keratinization in the hair follicle. (**A**) Keratinization of the outer root sheath (ORS). At the infundibulum level, keratinocytes of the hair follicle undergo the same process of cornification as that in the epidermis. Keratinocytes produce filaggrin, which organizes keratin intermediate filaments (KIFs) into tight, oriented bundles. The enzymatic cross-linking of the structural proteins and KIFs results in the CCE formation. TGM3 cross-links loricrin and small proline-rich proteins (SPRs) together in the cytoplasm of granular cells. (**B**) Keratinization of the inner root sheath (IRS). IRS cells produce trichohyalin, which is a functional analog of filaggrin. Trichohyalin becomes cross-linked to itself and—via multiple complex cross-linking—to other structural proteins providing mechanical strength to IRS and supporting the hair shaft growth. TGM3 has been suggested to be the main player in the cross-linking of trichohyalin. (**C**) The keratinization of cortex and cuticle. The hair cortex and the cuticle of the hair shaft are characterized by the expression of the diverse repertoire of keratins and keratin-associated proteins (KAPs). Within the cortex and the cuticle of the hair shaft, KAPs behave as trichohyalin by building the protein matrix that holds together KIFs. TGM3 participates in the cross-linking of the keratins and KAPs promoting the scaffolding of the hair shaft. (**D**) Keratinization of medulla. Medulla cells produce trichohyalin granules. However, due to the deficiency of KIFs, trichohyalin of the medulla cells is cross-linked mainly to itself during differentiation, resulting in the development of large vacuolated spaces. The process is mediated by TGM3.

The formation of CCE in the hair follicle occurs in the upper portion of the outer root sheath (ORS), in the inner root sheath (IRS), and in the hair shaft [27,61,62] (Figure 2). ORS is an external layer of the hair follicle. It may be considered as the invagination of epidermis, and it is not involved in hair formation. The upper part of ORS (the infundibulum) is a continuation of the interfollicular epidermis and undergoes a similar process of cornification as that in the epidermis [63]. The lower portion of the ORS consists of immature keratinocytes and does not produce cornified layers [64].

The formation of the hair follicle is initiated in the base of the hair bulb, where the highly proliferative matrix cells similar to the basal cells of the epidermis are located. When the matrix stem cells divide, they give rise to the intermediate populations of daughter cells, which continue to divide while they migrate upward toward the skin surface [65]. Here, their differentiation begins, and the concentric cell layers of the hair follicle are formed. While the basal cells of the interfollicular epidermis are the progeny of only one cell type, the matrix cells of the hair bulb produce many epithelial cell types ultimately differentiating into seven cell layers of the hair follicle [66]. Internally to ORS, the companion layer is located followed by three layers of IRS called the Henle’s layer, Huxley’s layer, and IRS cuticle, and three innermost layers called the cuticle, cortex, and medulla that form the hair shaft [67,68]. Single-cell RNA sequencing revealed the cells of a companion layer to be transcriptionally more similar to ORS than the inner layer cells [69]. Interestingly, there are three major cell clusters delineating the IRS, cortex/cuticle, and medulla layers, instead of the two for IRS and hair shaft, as one might suggest [69].

The keratinization of IRS begins with Henle’s layer followed by the synchronized keratinization of cuticles of IRS and hair fiber [70]. The process of IRS cornification is different from the one occurring in the interfollicular epidermis or the hair fiber. The keratinization of the cuticle and Henle’s layer of IRS progresses up to the Adamson’s fringe, the area where the hair shaft begins. Huxley’s layer is the last to cornify [70,71]. The cornification of IRS cells begins soon after they undergo differentiation from the matrix cells and the production of keratins and trichohyalin starts [70]. It mediates the keratin filament assembly and is initially accumulated in trichohyalin granules (like filaggrin in keratohyalin granules), which are unique in the inner IRS and medulla of the hair follicle [69,72]. Upon terminal differentiation, trichohyalin is subjected to posttranslational modification by peptidylarginine deiminase (PAD). PAD converts its arginine residues to citrullines and induces granule solubilization, making trichohyalin prepared for subsequent cross-linking by TGMs [72,73,74]. Trichohyalin becomes cross-linked to itself and—via multiple complex cross-linking—to other structural proteins, providing mechanical strength to IRS and supporting hair shaft growth [26]. More than 20 different proteins are implemented in the formation of the CCE of IRS, including SPRs, involucrin, desmoplakin, repetin, and epiplakin. This meshwork of proteins is assembled by TGM1 and TGM3 [26], but TGM3 has been suggested to be responsible mostly for the cross-linking of trichohyalin and KIFs compared to TGM1 [26].

The cornification of the medulla of the hair shaft occurs similarly to IRS in that medulla cells also produce trichohyalin granules. However, medulla cells contain few (if any) KIFs [72]. Due to the deficiency of KIFs, trichohyalin of the medulla cells is cross-linked mainly to itself during differentiation resulting in the development of large vacuolated spaces [72]. Such air-lifted spaces may contribute to thermal regulation in mammals [75]. The process is also mediated by TGM3 [72].

TGM3 was shown to make a substantial contribution to the later stages of keratinization in the hair cortex. The hair cortex and the cuticle of the hair shaft are characterized by the expression of the assembly of keratins and keratin-associated proteins (KAPs) [76]. Within the cortex and the cuticle of the hair shaft, KAPs behave as the trichohyalin of medulla and IRS [77] by building the protein matrix that holds together KIFs. Cortex cell differentiation is accompanied by the switch in keratin expression during the migration of cells in the direction from the lower to the upper parts of the cortex. First, keratins K35 and K85 begin to be expressed in less differentiated cells present in the lower part of the cortex, followed by the expression of keratins K32, K36 and K31 [76]. The expression of KAPs is delayed and occurs in more differentiated cells present in the upper part of the cortex. TGM3 participates in the cross-linking of the keratins and KAPs promoting the scaffolding of the hair shaft [76]. The expression of TGM3 coincides with keratins K31, K33-a, K33-b, K34, K83 and K85, and KAP2.n, KAP3.1, KAP3.3, KAP11.1, and KAP13.1 [76]. Cross-linking between the keratins and KAPs is preceded by the incorporation of disulfide bridges stabilizing KIFs [78].

Notably, TGM3 is the only TGM expressed in all three parts of the hair follicle (IRS, medulla and cortex), as evidenced by the recent single-cell RNA-seq analysis in mice [69]. TGM6 was shown to be expressed in the IRS and medulla, and TGM1 only in IRS [69] (Table 2). The abundant expression of TGM3 in all hair follicle layers supports the notion of its major contribution to the formation of cross-links in the hair shaft.

Expression of TGM3 plays an important role in epidermis differentiation in embryogenesis [32]. Akiyama et al. investigated the formation of CCE in a human fetus [79]. They showed that the expression of TGM1 and TGM3 along with CCE precursors loricrin and involucrin can be detected in the hair canal, IRS, and isthmus region of the ORS already at the bulbous hair peg stage, implicating TGM1 and TGM3 in the early stages of hair follicle development [79].

### 2.3. TGM3 Affinity to Substrates and Suggested Functional Compensation by Other TGMs

Several investigators identified TGM3 substrates in in vitro experiments. The activity of TGM3 was shown to be directed at the following proteins: SPR2, SPR4 [80], recombinant suprabasin, which is found in the interfollicular epidermis and the companion layer [69], and SPINK 6 expressed by cortex cells of the hair follicle [69]. All these proteins may be involved in epidermal differentiation [81,82]. Hornerin, a component of the interfollicular CCE and a protein of the S100 family, has also been reported to be a substrate of TGM3 at a late stage of CCE formation [83]. The presence of head A and head B domains suggests that SPR4 might be cross-linked by TGM3 and TGM1, respectively [52].

In vitro experiments showed TGM1, TGM3 and TGM5 to have similar affinity with loricrin, involucrin, and SPR3 [84]; however, all these enzymes demonstrate a specific cross-linking pattern, utilizing different residues for cross-linking and generating different products as a result of cross-linking [84].

Of great importance is the ability of different types of TGMs to compensate each other, which should be taken into consideration during the research practice. Most tissues express a combination of TGM isoforms, and some TGMs share the same substrate activity suggesting their complementary function. *Tgm2*-deficient mice (in spite of the abundant involvement of TGM2 in many processes) are viable and do not show serious abnormalities. It may be explained by the compensation of their functions by other TGMs. For example, it was shown that the expression of FXIIIa, TGM1, and TGM3 increased in bones and tendons in *Tgm2*-knockout mice [85]. *Tgm3*-deficient mice are also viable and do not show serious abnormalities. However, other TGMs (TGM1, TGM2, TGM5, TGM6) were shown not to change their expression pattern in *Tgm3*-deficient mice [86]. Thus, the mechanisms of TGM3 compensation in TGM3 mutant mice remain unclear.

### 2.4. Mouse Models for Studying the Role of TGM3 in Skin Morphogenesis

Animal models are a vital experimental tool in nearly all areas of biomedical research. Understanding the genetic mechanisms underlying biological processes becomes possible with the analysis of protein dysfunction or loss in mutant mice. Mice bearing spontaneous mutations along with engineered mutant mice have been characterized in TGM3 function research.

A thorough investigation conducted by John et al. to assess the role of TGM3 in the development of hair and the skin using *Tgm3*^−/−^ mice revealed the similar hair abnormalities as in people suffering from uncombable hair syndrome (UHS) [86]. Vibrissae of *Tgm3* mutant mice were twisted and thinner than in wild-type, pelage and tail hair showed a wavy pattern, which was most obvious in the first four weeks, and then, as in humans, the phenotype improved in a greater degree. Scanning electron microscopy showed that many hairs in *Tgm3*^−/−^ mice had a highly distorted cuticle, which was poorly retained upon the underlying cortex. The mild heating of hair in a reducing solvent led to loss of the cuticle. Transmission electron microscopy showed that the subcuticular zone failed to form normally, suggesting the hair cuticular cells to be directly compromised. Furthermore, Huxley’s layer, which has a high expression of TGMs including TGM3 [26], appeared fragile as well as corneocytes. In contrast to the abnormalities observed in hair development, no major defects were found in the interfollicular epidermis. Also, no overt changes in the barrier function of the skin indicated by normal transepidermal water loss, and toluidine blue and lucifer yellow dyes penetration were seen in *Tgm3*^−/−^ mice at birth. The same was observed for the ability to heal wounds. Interestingly, despite the widespread expression of TGM3 in the tissues of normal animals [31], its absence in the experimental mice did not cause severe malformation. Furthermore, *Tgm3*^−/−^ mice had a normal lifespan, were fertile, and produced litters of the expected size. Based on these findings, the authors state that TGM3 is highly significant in hair development and in particular, the cuticle, where it appears to have a unique role in stabilizing the trichohyalin network, whereas in stratified epithelia the loss of TGM3 is largely compensated.

It should be noted that in fact the barrier function of the skin in mutant *Tgm3*^−/−^ mice was altered to a higher degree than it was proposed by John et al. Following studies showed a higher permeability of the skin lacking TGM3. For example, more invasive percutaneous hapten FITC penetration from the skin surface followed by two-photon microscopy demonstrated a clinically latent skin barrier defect and indicated a reduced inflammatory threshold [87]. According to Frezza et al., in the absence of TGM3, the mouse skin also had reduced ultraviolet B (UVB)-filtering capability because the cornified layer presented altered composition and stability [88]. UVB radiation penetrated more deeply through the skin surface of *Tgm3* knockout mice, causing higher levels of DNA damage. The consequent apoptosis of the affected sun-burned cells was detected not only in the basal and suprabasal layers but also in the underlying dermis.

The crucial role of TGM3 in the maintenance of skin barrier integrity has also been highlighted in the recent studies of Piro et al., 2020. *Tgm3*^−/−^ mice generated impaired epidermal structure with more pronounced sensitivity than the normal skin to the psoriasis-inducing drug imiquimod. The authors observed anomalies in the spinous and granular layers of epidermis, suspecting incomplete keratinocyte differentiation and the defective cross-linking of loricrin [89].

The spontaneous mutation *wellhaarig* (*we*) was described in 1942 by Hertwig [90]. In 1990, Konyukhov et al. demonstrated *we* mice to have a wavy phenotype with hair abnormalities related to the keratinization of IRS [91]. *Tgm3* gene that causes the *we* phenotype is mapped on mouse Chromosome 2. Three variants of spontaneous defects in *Tgm3* are currently specified [92]. One of them shows a nonsense mutation in Exon 13 replacing cytosine with thymine, which leads to the emerging of a premature stop codon and the shortening of the mutant protein product TGM3 by 36 amino acids. *We^Bkr^* mutant demonstrates a missense mutation in Exon 7 replacing polar serine with nonpolar leucine and thus altering the catalytical core of a protein. The *we^4J^* allele is characterized by a 7 bp deletion in Exon 10, which also leads to a premature stop codon and shortened by 181 amino acids protein product. The *We^Btlr^* mutation, also known as tortellini mutation, corresponds to a G to A transition at Intron 3 eliminating Exon 3 (encoding 80 amino acids) from splicing [92]. All DNA defects result in altered and likely nonfunctional proteins.

The mechanism by which *Tgm3* mutation results in wavy hair is not fully understood. There is a possibility that the wavy phenotype develops due to the asymmetric cross-linking of the proteins in the hair cortex [92,93]. The abnormal cross-linking in the medulla seems not to contribute to the determination of the hair shape [94].

In addition, it is noteworthy that the gene *Tgm3* cooperates with the gene *waved alopecia* (*wal*), which is unmapped to date. Double mutant mice *Tgm3^−/−^wal^−/−^* develop the alopecia phenotype [95,96].

## 3. Contribution of TGM3 to Pathology

### 3.1. Structural Role of TGM3 in Disease: Uncombable Hair Syndrome

By now, the only prominent disease caused by the defect in TGM3 and connected with its structural role is UHS, also known as “spun glass hair syndrome”, “cheveux incoiffables” or pili trianguli et canaliculi. It was first described in 1973, but obviously, people had taken notice of it long ago. It is a very rare disorder characterized by the scalp hair being frizzy, wiry, dry, fair, standing away from the scalp in different directions and unable to be combed flat. It often has a spangled or glistening appearance due to light reflection from flattened and grooved hair surfaces [97]. However, the hair is not more fragile or brittle than normal hair, and the body and face hairs are not affected at all. In more than 50% of examined hairs, cross-sectioning reveals a reniform, triangular or heart-shaped form compared to the circular or oval outlines of normal hairs, as well as longitudinal grooves along the entire length of the hair shaft [97]. The clinical diagnosis of UHS can be confirmed by the scanning electron microscopy analysis of hair shafts [97,98,99]. No proven therapy exists for the disorder, but luckily, it is manifested in childhood and spontaneously disappears with age. Increased hair length improves the manageability of the abnormal hair, and hair entanglement is reduced in adolescents [42,100]. The majority of cases are inherited in an autosomal dominant manner with either complete or incomplete penetrance, and sporadic cases sometimes also take place [42,98,100,101]. UHS is mostly an isolated condition of the hair, but it has occasionally been observed with additional symptoms, such as loose anagen hair syndrome, ectodermal dysplasias, retinopathia pigmentosa, and juvenile cataract [102,103,104,105].

Further investigations in men expanded the understanding of molecular mechanisms of UHS development [42]. Recessive mutations in proteins involved in hair shaft formation PAD3, trichohyalin, and TGM3 were found in 11 children with UHS. It had been established before that trichohyalin is a structural protein co-localized with the PAD3 in the IRS of the hair follicle and in the medulla of the hair shaft [26]. Deimination by PAD3 reduces the overall charge of trichohyalin, and that enables its association with the KIFs. Then, trichohyalin and KIFs are cross-linked together by TGM3. KIFs are then stabilized, hardened, and linked to the CCE through further cross-linking by TGMs, particularly by TGM3 [26,72]. Based on this data and on their own findings, the authors identify UHS as the hair phenotype related to alterations not only in TGM3 but also in the other proteins of the trichohyalin-PAD3-TGM3 cascade. They draw the similar conclusion as was made by John et al.: the fact that no anomalies have been reported in the interfollicular epidermis of individuals with isolated UHS as in stratified epithelia of *Tgm3*^−/−^ mice [86] may be explained by the presence of other isoforms of these enzyme families in the epidermis, which can compensate for the loss of PAD3 and TGM3 activity, whereas these two are the only isoforms detected in the human hair cuticles and medulla [26,73,106]. They also speculate that the improvement in UHS observed with age might be either due to the compensatory expression of another isoform of PAD, TGM, or other structural hair shaft components or to mechanistic influences of aging-related changes in hair follicles, such as the increase in the diameter and length.

It is logical to assume that TGM3 participates in hair shape determination and may influence hair curvature as a result of its deficiency or asymmetric cross-linking of keratins to KAPs. A missense variant of TGM3 which affects the catalytic core of the protein enzyme contributes to African hair texture [107].

### 3.2. Functional Role of TGM3 in Disease: TGM3 as a Tumor Marker

Recently, articles began to appear that emphasized the role of TGM3 as a marker of different types of epithelial cancer. TGM3 was shown to be either downregulated or upregulated in various carcinomas (Table 3). In most studies, the reduction or loss of TGM3 expression was significantly associated with cell dedifferentiation and proliferation, increased invasiveness, and high incidence of lymph node metastasis, hematogenous recurrence and poor patient prognosis. More recently investigators began to look for the mechanisms guiding these processes and regulating TGM3 expression. First of all, they found out that the hypermethylation of CpG islands within the TGM3 promoter is involved in its repression, and histone deacetylation does not contribute to the transcriptional silencing of TGM3 [34,35]. He et al. also reported that the loss of heterozygosity within and near the *Tgm3* gene might lead to the downregulation of TGM3 in laryngeal carcinoma [108]. Furthermore, it was proposed that TGM3 downregulation may be involved in decreased rates of apoptosis in tumors. Several studies made on tumor cell lines indirectly demonstrated that the suppressive effect of the TGM3 protein on cell proliferation was caused by apoptosis and not by the alteration of the cell cycle. Wu et al. showed that ectopic TGM3 expression reduced the protein levels of full-length PARP, procaspase-3, procaspase-8, and the inhibitor of apoptosis Bcl-2 and increased the protein level of cleaved PARP and pro-apoptotic marker Bax in head and neck squamous cell carcinoma lines HN4, HN13, and HN30 [35]. The ectopic expression of TGM3 in esophageal cancer cell lines, such as SKGT-4, KYSE-510, OE33, and OE21, decreased cell proliferation and induced apoptosis by the modulation of the nuclear factor kappa-light-chain enhancer of activated B cells (NF-κB) signaling pathway [109], whose upregulation is known to inhibit apoptosis, promote cell proliferation, metastasis, metabolic changes, and other abnormalities that favor the expansion and spread of malignancy [110,111].

Recently, new data appeared suggesting that TGM3 may play a role in the propagation of cancer by modulating epithelial-to-mesenchymal transition (EMT) in epithelial carcinomas. This was indirectly proven by studies in colorectal cancer cell lines HCT116 and LoVo by assessing the expression of classical EMT markers [36]. The overexpression of TGM3 in these cells increased the expression level of the epithelial cell marker E-cadherin [119] and decreased the levels of mesenchymal cell markers N-cadherin and vimentin [120], while the knockdown of TGM3 reversed these processes. Besides, it was shown that the inhibition of EMT by the overexpression of TGM3 was conducted through the suppression of the PI3K/AKT signaling pathway, which is known to mediate the process of EMT and has attracted widespread attention as a potential target for the prevention and treatment of metastatic tumors [121,122].

Smirnov et al. conducted a study to reveal alterations of TGM3 expression in different types of skin cancer comparing with its expression in the layers of normal skin where each particular tumor originates from [118]. In agreement with the results mentioned above, TGM3 was downregulated in aggressive squamous cell carcinomas (SCC) and even absent in poorly differentiated ones, while it showed localization in the upper spinous and granular layers of normal skin [44,123]. Melanoma as well as melanocytes was negative for it [124]. Surprisingly, the upregulation of TGM3 was observed for basal cell carcinoma (BCC) comparing with the absence of its expression in the corresponding basal cell layer of normal skin [125]. Furthermore, at the mRNA level, the expression pattern of TGM3 was crucially altered in BCC but not in other types of skin cancer. Though the authors did not suggest any molecular mechanism for the downregulation of TGM3 in BCC, they proposed that the differentiation-correlated profile, including the correlation of expression between TGM1, TGM3, TGM5, loricrin, and involucrin, is lost in BCC in contrast to normal skin, SCC, and melanoma that was shown using the gene array. Another group of authors, who discovered the connection between TGM3 gene sequence alterations and BCC, hypothesized that compromised TGM3 activity might disrupt the normal differentiation and cell death program of corneocytes and the formation of CCE affecting skin barrier function, which in combination may cause inflammation leading to epidermal hyperplasia and the creation of tumor-promoting environment [126]. Thus, these two scientific groups contemplate the disturbance of the structural function of TGM3 in the propagation of BCC. However, taking into account the functional role of TGM3 in the development of different epithelial tumors, one can think that they may be partly right or even wrong, if only it does not participate in distinct pathways of cancerogenesis in the basal cell layer of the skin epidermis and in other epithelia.

Even more interesting data were published by Hu et al. [117]. TGM3 appeared to have prognostic significance in hepatocellular carcinoma (HCC). Moreover, its expression was much higher in transformed cells compared to normal liver tissue. The upregulation of TGM3 in HCC was demonstrated to make a similar contribution to the pathogenesis of HCC as was shown for its downregulation in head and neck squamous cell carcinoma, esophageal, and colorectal cancer. For example, TGM3 depletion in tumor cell lines Huh7 and 97H led to diminished cell proliferation, increased levels of cleaved caspase 3/apoptosis, and decreased colony formation, thereby contributing to decreased tumorigenesis and invasion. The results of this study suggested that TGM3 was involved in the positive regulation of multiple oncogenic pathways, including the PI3K/Akt, MEK/ERK, and NF-kB, which led to the increased cell survival and metastasis of HCC. Besides, TGM3 appeared to control EMT programming in HCC as was demonstrated by the downregulation of vimentin, fibronectin 1, and N-cadherin and the corresponding upregulation of E-cadherin in Huh7 and 97H cells with TGM3 knockdown. The authors speculated that these effects were most likely caused by the activation of the AKT/GSK3β/β-catenin signaling axis.

Thus, we have some understanding of the crucial functional role of TGM3 which is resolved in the development of epithelial cancers, but many elements, such as the upstream stimuli that lead to the altered regulation of TGM3, or the reasons why not only the downregulation but also the upregulation of TGM3 in certain tumors lead to similar consequences, need further investigation. Besides, almost all investigators (except those studying skin cancers) look at the problem of cancer development from the point of view of a molecular biologist, as we did not find any mentioning of the origin of cancer in each case. It seems to be equally important to address the problem from the point of view of a developmental biologist or a histologist to obtain an understanding of exactly which cells are changing and giving rise to the tumor. Other more interesting questions with regard to this are why neither people nor mice with TGM3 mutations having hair development abnormalities were shown to have epithelial malignances and how the structural (enzymatic) and functional roles of the enzyme cooperate in the tissues.

### 3.3. The Role of TGM3 in Development of Dermatitis Herpetiformis, Atopic Dermatitis and Oral Lichen Planus

One more disease, in the manifestation of which TGM3 occasionally participates, is dermatitis herpetiformis (DH). A very comprehensive review devoted to DH was recently published by Antiga et al. (2019) [127] and covers all the scientific and medical data collected to date concerning its history, epidemiology, pathogenesis, diagnosis, and treatment. Therefore, we will only briefly address the basic facts. DH is an autoimmune inflammation of the skin presented by the symmetric distribution of polymorphic papules, vesicles, and blisters on the extensor surfaces of the major joints as well as on the buttock, face, ears, neck, scalp, and groin accompanied by the pruritus, which is usually very annoying [128]. It is a common extraintestinal manifestation of the small bowel chronic disorder celiac disease (CD) and is characterized by granular IgA deposits in the papillary dermis confirmed by immunofluorescence biopsy [129]. Both CD and DH are associated with cereal gluten sensitivity in men [130]. The mechanisms of DH development include the manifestation of CD in the gut. Thus, patients with DH have gastro-intestinal symptoms similar to patients with CD. Most frequently, they are diagnosed with villous atrophy and seldom only minor enteropathy [131]. At the molecular level, in the course of CD, TGM2 deamidates gliadin proteins contained in gluten, which leads to the formation of immunogenic epitopes [132]. They are often recognized as antigens by antigen presenting cells, which possess the alleles HLADQ2 and HLA-DQ8 of the major histocompatibility complex class II expressed manly in Caucasians [133] causing an adaptive immune reaction against both TGM2 and gliadin [134,135]. Then, the phenomenon of epitope spreading most probably occurs because of the high sequence homology between TGM2 and TGM3 [136], and IgA autoantibodies begin to be produced in the small bowel also against TGM3 [43,137]. Finally, immune complexes of high avidity IgA with TGM3 are deposited in the papillary dermis. The mechanisms leading to tissue damage in DH are only partly understood, but the key role in DH inflammation is given to the neutrophils. All patients with DH as well as with CD are prescribed a life-long gluten-free diet. Unfortunately, even with the diet, IgA-TGM3 aggregates in the skin disappear very slowly [138]. Thus, cutaneous manifestations may last months or even years after the introduction of a gluten-free diet. Therefore, in most cases, pharmacologic treatment is required in order to control itching and the skin rash [139].

TGM3 was also revealed as an autoallergen in atopic dermatitis (AD) and was demonstrated to be actively involved in skin inflammation in this condition [140]. The mechanisms of skin inflammation in the case of DH and AD are most likely to be different as the authors showed that anti-TGM3 IgG but not IgA or IgM was increased in AD. They proposed the following role of TGM3 in the pathogenesis of AD. Th2 cytokines and/or allergen increase the expression of TGM3 in keratinocytes in the lesioned skin of AD patients, and tissue damage and exogenous agents lead to the release of intracellular TGM3. Then, TGM3 is presented to monocyte-derived dendritic cells via dendritic cell-specific ICAM-3-grabbing non-integrin, which activates the NF-kB signaling pathway in them, leads to the production of IL-6, and induces Th1 cytokine responses. IFN-g produced by T cells participates in keratinocyte damage, contributing to the chronicity of the disease. However, the exact role of TGM3 in AD remains unknown and needs further investigation.

To finalize the list of disorders developing with TGM3 involvement, we have to mention the disease named oral lichen planus, which is the type of mucositis. The abnormal localization of TGM3 (membranous instead of cytosolic) allowed the authors to suggest the contribution of TGM3 to hyperkeratinization frequently occurring in this condition [141]. They showed that involucrin localization on the plasma membrane and altered distribution of TGM1 and TGM3 in the keratinocytes of mucosa lead to keratinization, thus changing the mode of non-keratinizing squamous epithelium to the keratinizing one.

## 4. Conclusions

All of the above data suggest that TGM3 is highly significant in the development of the epidermis and the hair being particularly essential for the formation of CCE and the hair cortex, where it appears to have a unique role in stabilizing the protein network. However, it is likely that TGM3 is not critical for global body physiology and pathology since *Tgm3*^−/−^ mice, as well as people with Tgm3 mutations, show a normal phenotype with abnormalities mostly related to the hair shaft shape. This probably reflects the compensational role that can be exhibited by other isoforms of TGM due to their affinity to the same substrates. High sequence homology between TGM2 and TGM3 also contributes to DH development in people with gluten sensitivity. It is interesting to note that TGM3 also has other functions that are not related to protein cross-linking, such as participation in cancerogenesis. Either the upregulation or downregulation of TGM3 expression in different kinds of epithelia (not only epidermis) is thought to be crucial for the activation of multiple oncogenic pathways and the modulation of EMT causing cell dedifferentiation, increased cell survival, and metastasis, and thus poor prognosis for patients. Implication in pathology makes this enzyme of a particular interest as a carcinoma marker and deserves further investigations as too many questions remain unanswered.

## Figures and Tables

**Table 1:** Skin-related TGMs.

Type of TGM	Synonyms	Function	Human Skin Diseases	Knockout Mouse Models	References
TGM1	Keratinocyte TGM	Cornified cell envelope (CCE) formation, wound healing	Ichthyosis (lamellar ichthyosis, non-bullous congenital ichthyosiform erythroderma)	Defective stratum corneum and early neonatal death	[16,37,38,39,40,41]
TGM2	Tissue TGM	Apoptosis, wound healing, angiogenesis, matrix stabilization, cell differentiation	Associated with various human disorders, including inflammation, cancer, and fibrosis, a mediator of the epidermal inflammatory response to UV irradiation	TGM2 null mice appear normal; upon UV exposure, display decreased skin inflammation compared to that of wild-type mice; *Tgm2*^−/−^ fibroblasts demonstrate impaired adhesion in culture	[17,18,19,20,21,22]
TGM3	Epidermal TGM	CCE formation, hair fiber stabilization	Uncombable hair syndrome, dermatitis herpetiformis	Impaired hair development	[42,43,44]
TGM5	TGM X	Epidermal differentiation, CCE formation	Skin peeling syndrome, also involved in the hyperkeratosis in ichthyosis and psoriasis patients	No skin defects	[12,45]
TGM6	TGM Y	Late stage CCE formation in the epidermis and the hair follicle	No skin defects	No skin defects	[12]

**Table 2:** Distribution of TGMs in the epidermis and the hair follicle.

Type of Transglutaminase	Epidermis	Hair Follicle	References
TGM1	Granular layer	Three layers of IRS. The innermost layer of ORS in the distal part (close to isthmus) of ORS	[27,62]
TGM2	Basal layer	Hair germ and IRS of the bulbous hair peg	[19,79]
TGM3	Upper granular layers	Cortex, medulla, cuticle, IRS, companion layer	[23,27,69]
TGM5	A gradient of concentration from the basal layer to the stratum corneum	All three IRS layers, and residual quantities in the hair cuticle and the hair shaft, outer bulge, hair germ	[23,27,69]

**Table 3:** Regulation of TGM3 in different types of epithelial cancer.

Name of Cancer	Type of Epithelium	Type of Regulation	References
Oral carcinoma	Non-keratinizing stratified squamous epithelium	Downregulation	[34,112,113,114]
Laryngeal carcinoma	Non-keratinizing stratified squamous epithelium	Downregulation	[115]
Esophageal carcinoma	Non-keratinizing stratified squamous epithelium	Downregulation	[109,116]
Colorectal carcinoma	Simple columnar epithelium	Downregulation	[36]
Hepatocellular carcinoma	Simple cuboidal epithelium	Upregulation	[117]
Basal cell carcinoma (skin cancer)	Keratinized stratified squamous epithelium	Upregulation	[118]
Squamous cell carcinoma (skin cancer)	Keratinized stratified squamous epithelium	Downregulation	[118]

## Abbreviations

TGM	Transglutaminase
KIFs	Keratin intermidiate filaments
ORS	Outer root sheath
IRS	Inner root sheath
CCE	Cornified cell envelope
UHS	Uncombable hair syndrome
EMT	Epithelial-to-mesenchymal transition
BCC	Basal cell carcinoma
HCC	Hepatocellular carcinoma
DH	Dermatitis herpetiformis
AD	Atopic dermatitis
